# Configurations of the driving factors promoting China's commercial health insurance: A comparative qualitative analysis based on the technology–organization–environment framework

**DOI:** 10.1016/j.heliyon.2022.e11522

**Published:** 2022-11-11

**Authors:** Xiuquan Huang, Chih-Lin Tung, Xi Wang, Xiaocang Xu, Fat-Iam Lam, Tao Zhang

**Affiliations:** a Faculty of Humanities and Social Sciences, Macao Polytechnic University, Macao, 999078, China; b School of Economics and Management, Huzhou University, Huzhou, 313000, China

**Keywords:** Commercial health insurance, Qualitative comparative analysis, Configuration, Technology–organization–environment framework

## Abstract

With the nation's remarkable improvement in living standards, China's health insurance system cannot satisfy people's higher demands; therefore, it is necessary to promote the supply of commercial health insurance (CHI) in China. Based on the technology–organization–environment (TOE) framework, this study constructs a novel analysis framework to investigate the driving path of China's CHI. Employing the data of 31 provincial regions of China in 2018, a fuzzy-set qualitative comparative analysis is conducted to analyze configurations. We also adopt a necessary condition analysis in the robustness check to examine the necessary conditions, determining that no necessary relationship exists between possible conditions and the performance of CHI. More particularly, three sufficient configurations, TOE strategy, government attention (GA)–environment adaptability (EA)–citizen demand (CD) strategy, and dual EA–CD strategy are demonstrated to achieve high performance, and the other three configurations of technological management capability (TMC)–EA-CD strategy, technological infrastructure (TI)–EA strategy, and combined TI–TMC–EA strategy do not result in high performance. In addition, technological conditions (TI and TMC) and EA are relatively more important than the other configurations. Notably, government departments' financial expenditure is found to have a negative effect on CHI promotion.

## 1 Introduction

The uncertainty of the occurrence of disease is one of the most significant risks for humankind, particularly for those living in low-income households [1]. To manage health risk, households can save money for potential future health expenditures [2, 3, 4], borrow privately or from a bank [5], restructure jobs [6, 7], and purchase relevant insurance [8, 9]. As a stabilizer of social life, insurance is the most effective tool of risk management [10]. In 2000, the Chinese government established a universal medical insurance system, including Urban Employee Basic Medical Insurance (UEBMI), Urban Resident Basic Medical Insurance, and the New Cooperative Medical Scheme (NCMS) [11]. According to the China Statistical Yearbook 2011, universal medical insurance, covering 95% of the total population in China by 2010, has dramatically improved residents’ ability to withstand disease.

The medical insurance design under China's social security system is based on overall social and economic characteristics and does not offer adequately detailed options for individual citizens [12, 13]. Given the improvements in Chinese living standards, the basic medical insurance system is unable to meet people's diversified and high-level needs [14]. The World Health Organization (WHO) in 2011 projected that the elderly ratio in China would reach 27% by 2050, presenting another challenge to the Chinese health insurance system [15]. In mature insurance markets, such as those in America and Japan, the proportion of commercial health premium to life insurance premium income is about 30%. In comparison, this proportion is only around 20% in China, revealing a significant difference. Therefore, accelerating the development of commercial health insurance (CHI) is an essential approach to protecting citizens' health. In accordance with the enhanced market-oriented economic reforms [16], promoting the efficiency of the medical security system is a priority for the Chinese government [17].

In recent years, the Chinese government has issued several relevant policies to vigorously support CHI. Various CHI options are offered as an essential pillar of the social security system. In addition, with the deepened market-oriented reforms, the promotion of CHI must make full use of the market mechanism. Ongoing CHI development is thus affected by the government, the market, and other related factors. In the context of multiple factors, some important questions arise. Are there necessary factors for effective CHI development? What are the driving paths for CHI development? Are there other paths? This study answers these questions to aid the government's strategic formulation of more scientifically informed and accurate policies.

Many studies have examined the promotion of CHI. Lees and Rice [18] first theoretically analyzed the efficiency of the medical insurance market in the United States and investigated the demand for CHI among consumers with different risk attitudes. The study also constructed a framework to determine the expected utility function of CHI to express the choice of consumers with different risk attitudes and reveal optimal health insurance demand. Dardanoni and Wagstaff [19] studied whether national wealth affects the demand for CHI. Based on the uncertainty condition, the study established a net investment model, finding that national wealth significantly impacts the demand for CHI due to the introduction of uncertainty. Liljas [20] adopted an improved health demand model, taking the uncertainty of disease incidence as the basic assumption. The research results showed that residents’ health status and differences in health insurance products impact the demand for CHI. Doshmangir et al. [21] examined whether a series of health insurance reforms in Iran effectively promoted universal health coverage and the issues that must be prioritized to maintain sustainable universal health coverage.

The existing literature on CHI is extensive and particularly focused on the influencing factors of CHI promotion, which are divided into demand and supply. China's CHI is currently facing a good market environment [22, 23]. In addition, profit-seeking insurance companies calculate the prices for CHI using scientific actuarial techniques according to multiple objective variables, such as incident probability, treatment cost, and insurance fraud probability. The marginal cost of the insurance product is so tiny that the supply amount is unlimited; hence, the supply is not restricted, and its development is more driven by demand. Therefore, the relevant research on CHI promotion is primarily conducted from a demand perspective. The influencing factors of demand can be divided into factors related to purchasing intention and purchasing power [24, 25, 26, 27, 28, 29, 30, 31, 32]. The main factors affecting CHI promotion in supply are derived from relevant operational characteristics and agency insurance companies [33, 34, 35]. These factors are summarized in Table 1.

**Table 1:** Influencing factors of CHI promotion.

Demand	Factors related to purchasing intention	Past health experiences	Innocenti, et al. [24]
		Hygiene conditions	Asgary, et al. [25]
		Perception of risks	Chang, et al. [26]
		Health or life expectations	Acland and Levy [27]
		Social security system	Zhao [28]
		Residents' wellbeing	Zhao [28]
	Factors related to purchasing power	Price of CHI	Strombom, et al. [29]
		Household income	Hill, et al. [30]
		Taxation	Koo and Lim [32]
Supply	Relevant characteristics of operating and agency insurance companies	Role of insurance intermediaries	Sugawara and Nakamura [33]
		Differences in insurance products	Jensen, et al. [34]
		The proportion of state-owned shares	Nieizviestna, et al. [35]
		Company life	Nieizviestna, et al. [35]

Existing research has focused on the independent effects of single factors and does not consider the multi-factor synergistic effect of CHI promotion from the configuration perspective. In addition, a lack of research remains regarding the promotion of CHI from the perspective of technology adoption. As a means of risk management, CHI can be considered a technology to navigate risks, and purchasing CHI can be considered adopting and promoting this technology. Therefore, this study applies the fuzzy-set qualitative comparative analysis (fs/QCA) based on the technology–organization–environment (TOE) framework concerning technology adoption to examine the synergistic matching effect of multiple factors of CHI from the perspective of configuration. The research explores the configuration conditions and the driving paths of the promotion of CHI, filling the existing research deficiencies and providing a new analytical approach for the field of CHI. This is the first study to apply fs/QCA to analyze CHI promotion, using data from 31 provincial regions in mainland China in 2018.

## 2 Material and methods

### 2.1 A TOE framework for the promotion of CHI

Although there are numerous factors influencing the promotion of CHI, no widely accepted theoretical framework has emerged. The TOE theoretical framework has been widely applied in technology adoption in the configuration analyses [36]. The TOE framework has also been flexibly applied in many other fields by expanding the technical connotation. Some scholars have adopted TOE to explore the influence of these factors on Vietnamese firms’ innovation decisions [37]. Others have examined three antecedents and the influence of digital investment on IT innovation using the TOE framework [38]. Some have employed a multi-case study qualitative approach to explore the strategies used by e-retail microbusinesses to potentially advance e-business adoption [39].

**Figure 1:**
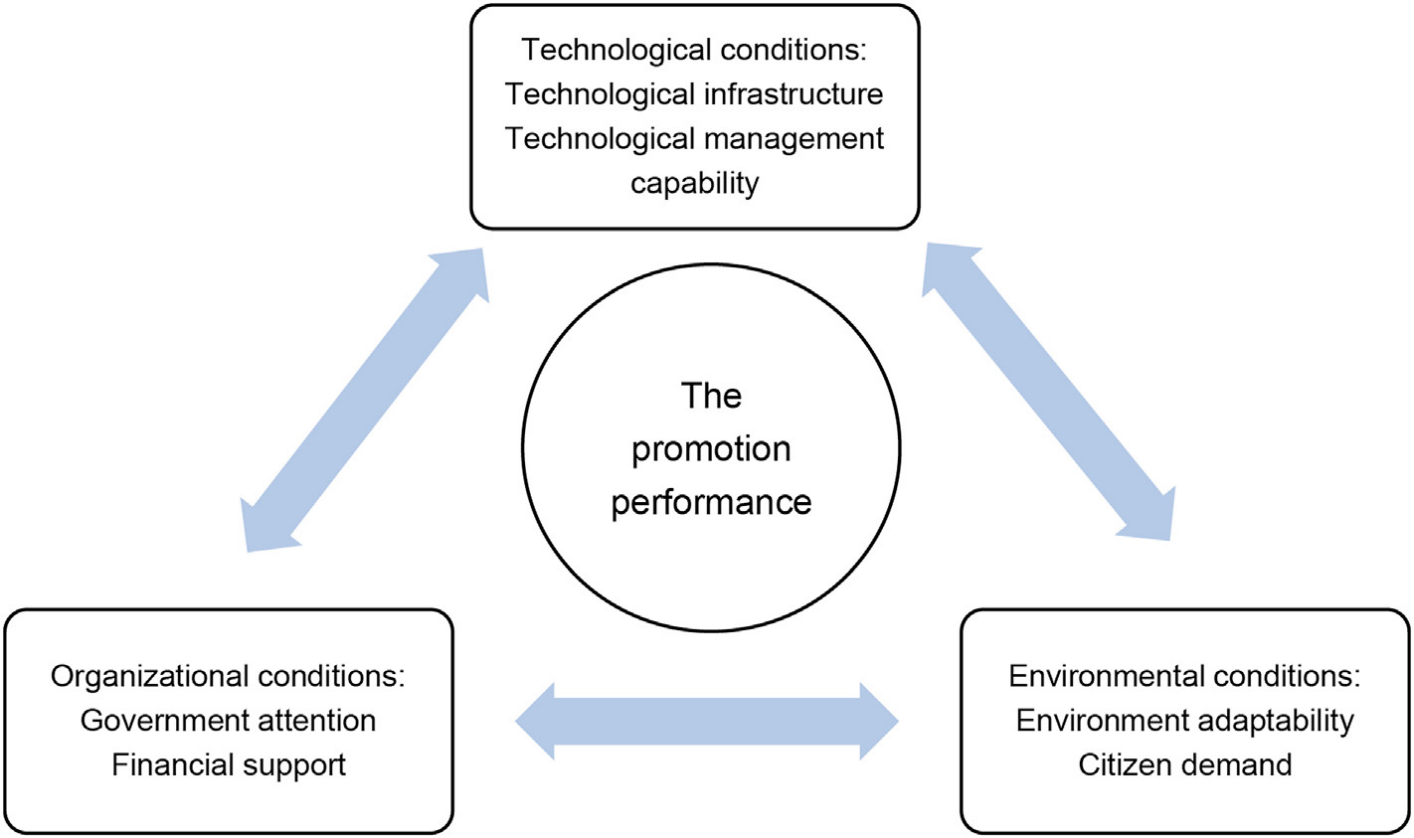
TOE framework for the promotion of CHI.

This study creatively introduces the TOE framework in technology adoption to examine CHI as technology, analyzing technology application and development levels. The TOE framework focuses on the multi-level and correlated impacts of technology, organization, and environment on technology promotion in society, providing a comprehensive analysis framework based on technology application scenarios. As a generic theory, the TOE framework can examine a variety of factors and freely change them according to the research problems and associated background, offering a wide range of applicability. The specific framework for analyzing the promotion of CHI constructed in this study is presented in Figure 1.

First, technological conditions include technological infrastructure (TI) and technological management capability (TMC). Technology factors primarily refer to the accessibility of technology and the ability to support innovation adoption, focusing on whether technology can match the organization and produce potential benefits. An organization's technology application ability establishes a wide range of restrictions and the possibility for technology change. TI, which is closely related to the efficiency of technology promotion, is similar to infrastructure in economic development, which has a crucial role in promoting the economy. TMC reflects technology promoters' resource allocation ability and affects promotion efficiency.

Second, organizational conditions include government attention (GA) and financial support (FS). Organizational factors are related to organizations' characteristics and resources, including the structure, scale, and quantity of redundant resources. The implementation of policies and projects in China is significantly affected by government departments’ degree of concern. The more attention paid by the government, the more administrative resources and support it receives, elevating policies to a higher priority. In addition, some FS, such as financial subsidies and tax preferences, can be helpful for technology promotion.

Third, environmental conditions include environment adaptability (EA) and citizen demand (CD). Environmental factors are structure, demand pressure, and the institutional environment. EA measures the acceptance of technology in the local social environment, and CD measures the potential market demand for technology. These two factors can measure the difficulty of technology promotion from the perspective of the existing environment.

### 2.2 Fuzzy-set QCA procedures, methods, and data preparation

The fs/QCA is a new method based on set theory that has emerged in recent years. It is suitable for configuration analyses and has been applied in many contexts. Details regarding the methodological approach can be found in Ragin [40]. Unlike other qualitative comparative analyses, fs/QCA can handle the problems of degree change or partial membership. For this reason, fs/QCA has been widely used in relevant empirical studies in recent years, employing the method to explain the public policy for innovative governance [41], to analyze the impact of surge pricing on customer retention [42], and to identify the configurations in the relationship between environmental practices and other management practices to improve labor productivity [43]. This study employed fs/QCA to analyze the multi-level and linkage matching impact on CHI technology promotion based on the TOE framework.

The fs/QCA method has four advantages for investigating CHI. First, case-oriented QCA analyzes problems from a comprehensive, whole system's perspective and focuses on how the antecedents and conditions combine to produce results. This method solves variables' interdependence and causal complexity through quantitative analyses, which cannot be accomplished by traditional quantitative analyses [44]. This is because traditional approaches emphasize the competition among variables, taking an isolated perspective when treating each variable. Although there is a moderating effect in traditional quantitative analysis for analyzing the effect of multiple conditions on the results, it strictly limits the number of conditions to no more than three [40]. Second, the QCA method uses Boolean algebra, avoiding errors due to the omission of variables in the model [45]. Third, QCA is suitable for small, medium, and large-scale samples, overcoming the shortcomings of qualitative and quantitative analyses [40]. Qualitative analysis is comprehensive and in-depth enough for case data, but the generality of results is insufficient. Quantitative analysis can draw general conclusions, but the information of individual cases lacks depth. Finally, for the high and not-high performance of the promotion of CHI, QCA can also examine causal asymmetry [46].

### 2.3 Measures and calibrations for set membership

Calibration is a common operational step in physics, astronomy, and other natural science research, in which natural science researchers adjust the instruments to calibrate the observed data to meet the corresponding standards in the discipline. Similarly, the calibration of original data is an indispensable step in QCA, using a method based on validated theory. Furthermore, uncalibrated data can only reveal the relative position of a variable. For example, this study can only determine whether one province has a higher urban rate than another according to the uncalibrated urban rates of two provinces, but whether the province belongs to highly urbanized provinces is difficult to judge. Specifically, both Beijing and Sichuan have higher urban rates than Tibet. This study can discern that Beijing is a highly urbanized province, while Sichuan is not only by using a standard of the highly urbanized province. Anchor points must be established for each variable according to theoretical knowledge. This study chooses three anchor points (fully out, crossover point, and fully in) and uses the logistic function to calibrate the original data to the values distributed between 0 and 1 [40].

This study employs the most used direct calibration method, which can overcome the lack of precise theoretical guidance and experience and subjectivity [47]. As shown in Table 2, the 75% and 25% quantiles of variables were used as the anchor points or thresholds to represent fully out and fully in designations, and the mean value of 75% and 25% quantiles as the crossover point.

**Table 2:** Sets and calibrations.

Sets	Conditions	Fully out	Crossover	Fully in
Outcome	Promotion performance	0.18	0.244	0.307
Technological conditions	Technological infrastructure	26	59	92
	Technological management capability	553.5	1000	1446.5
Organizational conditions	Government attention	1381	837	293
	Financial support	6.91	7.96	9.01
Environmental conditions	Environment adaptability	53.38	59.52	65.66
	Citizen demand	9.655	11.246	12.837

This study chooses some variables to represent these conditions, and Table 3 presents the variables’ basic summary statistics. This study measures CHI promotion performance using the insurance density of CHI that is equal to per capita premium income; the larger the index is, the wider the scope of insurance coverage is. This is positively related to promotion performance. Other indicators include insurance depth (premium divided by GDP), premium income, and some uncommon indicators, but scholars commonly use insurance density [48, 49]. This study assigns 0.18, 0.244, and 0.307 as the thresholds for fully out, crossover point, and fully in the promotion of CHI set.

**Table 3:** Summary statistics for all variables.

Variables	Mean	Sd	Min	Max	Number of observations
Healthcare insurance density	0.26753	0.16886	0.067162	1.016709	31
Insurance agents	78.16129	84.951	2	402	31
Number of students	1205.032	1009.608	270	4711	31
Implementation timing	690.8065	544.6109	32	1503	31
Healthcare expenditure proportion	8.049841	1.407126	5.426046	10.33027	31
Urban rate	59.9871	11.78289	31.14	88.1	31
Elderly proportion	11.26514	2.502227	5.679801	15.16236	31

This study measures TI using the number of professional insurance intermediaries. Insurance intermediaries are individuals and legal persons who specialize in the insurance business. The promotion of technology requires sales associates to connect the technology with actual demand. Driven by profits and professionalization, insurance intermediaries are the foundation of CHI promotion [33, 50]. The anchors 26, 59, and 92 are assigned as the thresholds for fully out, crossover point, and fully in for the TI set.

TMC is measured by the number of college students majoring in insurance. Education related to insurance determines professionalism, influencing the promotion efficiency of the technology; hence, the indicator reflects the investment and reserves of professional insurance human resources, which is closely related to the technical management ability of insurance operation [51, 52]. This study uses 553.5, 1,000, and 1,446.5 as thresholds for fully out, crossover point, and fully in the TMC set.

This study measures GA using the days that the provincial government took to issue corresponding implementation policy documents for the guidance document issued in November 2014 by the central government—*Opinions of the general office of the State Council on accelerating the development of commercial health insurance.* The document was a vital landmark document for promoting CHI, with most provinces and municipalities issuing subsequent documents within two years and Guizhou province as the latest in August 2018. It is reasonable to use December 30, 2018, as the deadline for this study because data calibration steps will not affect the scientific nature of the analysis results. The shorter the interval, the more attention the provincial government paid to the promotion of CHI; thus, in contrast to other indicators, the smaller the index is, the higher the membership score is. The thresholds for fully out, crossover point, and fully in the high GA set are 1,381, 837, and 293.

This study measures FS using the proportion of medical health and family planning support in the local general public budget to assess the behavior that people purchase CHI related to purchasing health and medical security services in the future [25]. The higher the expenditure on health care and family planning, the more superior the health and medical security services the insured can enjoy [53]. Indirectly, FS improves the value of CHI, which will affect the demand. The study assigns 6.91, 7.96, and 9.01 as the thresholds for fully out, crossover point, and fully in the FS set.

This study measures EA using the urbanization rate (the proportion of the urban population). EA is a comprehensive measure of the acceptance of CHI by all sectors of society. Generally speaking, the higher the urbanization rate, the more mature the CHI market [54]. As a consumer behavior, purchasing CHI is influenced by many aspects of society, and the urbanization rate is the comprehensive index used to measure acceptance [55]. This study assigns 53.38, 59.52, and 65.66 as the thresholds for fully out, crossover point, and fully in the EA set.

This study measures CD using the proportion of the elderly over 65 years of age in the total population. CD reflects the potential demand for CHI in the current environment; the higher the elderly population, the greater the demand for CHI [56, 57]. This study calibrates the scale anchors 9.655, 11.246, and 12.837 for fully out, crossover point, and fully in the CD set.

### 2.4 Analysis of sufficiency and necessity

QCA is primarily employed to analyze the sufficient or necessary conditions (in essence, set relations) between each condition and its configuration and results. Conducting the necessity analysis before the sufficiency analysis is beneficial to the counterfactual analysis during the configuration analyses. The key indicator for determining necessity is consistency. A condition can be considered necessary if the consistency is greater than 0.9 [58,59]. In the analysis of necessary conditions, consistency refers to the proportion of outcome contained by a condition, and the proportion of the intersection of outcome and the condition to the outcome. The three key indicators used to judge the sufficient configuration include consistency, the proportional reduction in inconsistency, and case frequency, the three critical values of which are 0.8, 0.75, and 1, respectively [60]. Consistency in sufficient condition analysis refers to the proportion of a condition contained by the outcome; that is, the proportion of the intersection of outcome and condition to the condition. Proportional reduction in inconsistency helps to identify the relationships of simultaneous subsets, meaning that one configuration is sufficient to both outcome and the negation of the outcome. Case frequency refers to the number of cases observed in a configuration. If the three indicators reach their respective critical values, the configuration and the outcome constitute a sufficient condition relationship.

If a configuration has no corresponding outcome or case, then that configuration is a logical remainder. The logical remainder does not mean that the configuration does not exist in real life, only that it is not observed in the sample. Researchers can speculate based on relevant knowledge to determine whether there is a sufficient relationship between the configuration and the result in reality. If relevant theories or practical experience show that a logical remainder constitutes a sufficient relationship with the result, this kind of logical remainder is called an easy logical remainder; otherwise, it is a difficult logical remainder. In the research, logical remainder can be introduced in the process of QCA analysis results. The logical remainder or configuration can be considered to have a sufficient relationship with the results, which is called counterfactual analysis. The results presented include three kinds of solutions: conservative, intermediate, and parsimonious. Conservative solutions are the results of QCA analysis that does not conduct the counterfactual analysis and does not contain any logical remainders, intermediate solutions are the results of the analysis that only introduces easy logical remainders in counterfactual analysis, and parsimonious solutions are those that introduce all logical remainders in counterfactual analysis.

Unlike most existing QCA literature, this study presents enhanced conservative, intermediate, and parsimonious solutions, removing unreasonable assumptions in counterfactual analysis, which is neglected in most existing studies [58]. The unreasonable assumption is not equivalent to a difficult logical remainder, and both easy and difficult logical remainders may contain unreasonable assumptions. There are three kinds of unreasonable assumptions used in this study. The first is that logic does not exist, such as a pregnant man; the second is that one configuration is the sufficient condition of the outcome and negation of the outcome; the third is the configurations that are the simultaneously necessary condition of the outcome and sufficient condition of negation of the outcome [61, 62].

The research results are presented following the form commonly adopted in the current literature using QCA. Specifically, the symbols ● and ⊗ represent the presence and absence of the core condition, respectively, while the smaller symbols, ● and ⊗, with the same shape represent the presence and absence of the peripheral condition. The presence and absence of one condition denotes that a condition should be high or low in the configuration, respectively. A blank indicates the presence or absence of a condition does not matter. Core conditions are those present in both parsimonious and intermediate solutions, whereas peripheral conditions are only present in intermediate solutions. Note that core and peripheral conditions are robust, and the difference is that the core condition is more robust than the peripheral one; therefore, the size of the symbol indicates the degree of significance. In the counterfactual analysis process, a researcher can set the directional expectation of each condition. TI, TMC, EA, and CD are set as the conditions for the outcome of high performance of promotion of CHI in this study. Due to the limitations of the Barrel Principle and lack of data calibration accuracy, the configuration of an outcome is complex; therefore, only EA is set for absence signaling the outcome of non-high performance, which is relatively conservative.

After obtaining all the configuration results, we can summarize them to analyze the substitution relationships between the conditions. There are often multiple valid paths to one result, and the difference between the conditions contained in these paths can reflect the potential substitution between the conditions. In particular, a substitution relationship is apparent when two paths have multiple conditions that are the same. This analysis is undoubtedly essential. For example, to achieve high CHI promotion performance, it is difficult for a province to prioritize a particular condition in a configuration highly for objective reasons. Based on the substitution relationship, a province can strategically formulate policies to induce other conditions to compensate for this non-high condition. This study essentially presents an analysis of the selection of the best configuration.

### 2.5 Necessary condition analysis (NCA)

This study adopts NCA, which is a rarely used method, to analyze the necessary conditions, strengthening the robustness of necessary conditions in QCA analysis [63]. QCA can identify the necessary relationship, but only qualitatively reveals whether a condition is necessary or unnecessary for an outcome. It does not quantitatively reflect the extent to which a condition is necessary for an outcome [64]. Therefore, the fuzzy-set change is not only a yes or no matter but also includes detailed membership scores, making the combination of NCA and fs/QCA more valuable. NCA can identify whether a specific condition is necessary for a certain outcome and analyze the size of the effect of the necessary condition, which is referred to as the bottleneck level, identifying the lowest level of necessary conditions to produce a specific result. The larger the effect size in the 0–1 range, the larger the effect is. In the NCA, the necessary conditions must meet two requirements. The effect size cannot be less than 0.1, and the Monte Carlo simulation permutation test of the effect size must be significant [65]. The study employs ceiling regression (CR) and ceiling envelopment (CE) to generate the upper bound function, comparing the two results for robustness.

### 2.6 Data source

This study uses provincial data from 31 provinces in China in 2018 for analysis. The development of China's commercial insurance is unbalanced among these 31 provincial regions. For example, according to the 2016 data issued by the China Insurance Regulatory Commission (CIRC), the top 10 provinces in premium income are predominantly in economically developed areas, with the total premium accounting for about 70% of the total. However, the remaining 30% of provinces' premium income accounts for less than 10% of the total. In addition, Chinese provinces' socioeconomic development is imbalanced. This phenomenon is beneficial for the observation of various combinations of conditions and meets the need for research. The data of this study are obtained from the *China Statistical Yearbook*, the *China Insurance Yearbook,* and the *China Stock Market & Accounting Research Database.*

## 3 Results

### 3.1 Analysis of necessary conditions

Tables 4 and 5 present the results of the necessity condition analysis in fs/QCA and NCA, respectively. Table 4 demonstrates that all consistencies are less than 0.9, indicating that all conditions and the negations of conditions do not constitute necessary conditions for high and not-high performance outcomes of CHI. Table 5 shows that no conditions can meet the two requirements in CE and CR except EA. However, EA's effect size using CE is 0.135, just slightly more than the threshold value of 0.1. Although the consistency in fs/QCA is slightly less than 0.9, this study conservatively asserts that the necessary conditions do not hold, demonstrating that the promotion mechanism of CHI is complex, and the explanatory power of a single factor is weak.

**Table 4:** Analysis of necessary conditions for high and not-high CHI performance in fs/QCA.

Sets of conditions	High performance		Not-high performance	
	Consistency	Coverage	Consistency	Coverage
TI	0.677	0.697	0.382	0.416
∼TI	0.433	0.398	0.722	0.703
TMC	0.666	0.681	0.39	0.422
∼TMC	0.435	0.402	0.705	0.691
GA	0.621	0.474	0.732	0.592
∼GA	0.466	0.621	0.35	0.494
FS	0.416	0.362	0.768	0.707
∼FS	0.663	0.729	0.308	0.358
EA	0.806	0.857	0.246	0.277
∼EA	0.32	0.286	0.873	0.826
CD	0.627	0.601	0.476	0.483
∼CD	0.46	0.453	0.606	0.632

Note: ∼ indicates the absence of a condition.

**Table 5:** Results of necessary condition analyses (NCA).

Condition	Method	Accuracy	Ceiling zone	Scope	Effect size	p-value
TI	CE	1	0.021	0.994	0.021	0.004
	CR	0.742	0.256	0.994	0.258	0.001
TMC	CE	1	0.029	0.992	0.03	0.013
	CR	0.871	0.023	0.992	0.023	0.08
GA	CE	1	0	0.961	0	1
	CR	1	0	0.961	0	1
FS	CE	1	0	0.998	0	0.009
	CR	1	0	0.998	0	0.008
EA	CE	1	0.135	1	0.135	0
	CR	0.871	0.291	1	0.291	0
CD	CE	1	0.003	0.999	0.003	0.01
	CR	0.806	0.112	0.999	0.112	0.006

### 3.2 Analysis of sufficient condition

This study analyzes the configurations of high and not-high CHI performance, respectively, presenting the results in Table 6. From the perspective of a single configuration, there are three configurations to achieve high and not-high performance. The consistencies of all configurations are greater than 0.9, higher than the set threshold of 0.8, indicating that the analysis result of every single configuration is reliable. Overall, the consistency of the three configurations to achieve high performance is 0.904, and that of the three configurations to achieve not-high performance is 0.975, both of which are higher than the generally accepted level of 0.8. The high and not-high performance coverage is 0.648 and 0.519, respectively, referring to the extent to which a result can be explained by configurations, similar to the determinable coefficient in econometrics.

**Table 6:** Configurations for achieving high and not-high CHI performance (fs/QCA).

Configuration	High performance			Not-high performance		
	1	2	3	4	5	6
TI	•			•	⊗	⊗
TMC	●			⊗		⊗
GA	•	•			•	
FS			⊗	●	●	●
EA	●	●	●	⊗	⊗	⊗
CD		●	●	⊗		•
Consistency	0.931	0.958	0.892	1	0.97	0.926
Raw coverage	0.319	0.293	0.42	0.11	0.433	0.164
Unique coverage	0.127	0.046	0.228	0.05	0.294	0.031
Overall solution consistency	0.904			0.975		
Overall solution coverage	0.648			0.519		

Note: ● = core causal condition present; ⊗ = core causal condition absent; • = peripheral condition present; ⊗ = peripheral condition absent; blank = the presence or absence of conditions does not matter.

Solution 1 reveals a valid *TOE* strategy*.* With four high conditions, it is the configuration with the most high-level conditions, and also has high conditions in all TOE aspects, representing the most comprehensive configuration and balanced path for high performance. Specifically, a combination of high TI, TMC, GA, and EA is sufficient for high performance. As shown in solution 1, the raw coverage (0.319) explains a high proportion of the high performance.

Solution 2 represents a *GA–EA–CD* strategy*.* High CHI performance can be facilitated by using high GA combined with high EA and high CD. High EA and high CD demonstrate that CHI operates in a positive environment and has considerable potential demand. Meanwhile, high GA indicates that government departments prioritize the growth of CHI in the process of technology promotion. As a market regulator, government has a significant promotional influence, as it can cooperate with the “invisible hand” to address market failure and act as an accelerator, formulate industry standards, and introduce policy guidance. The solution's raw coverage is 0.293, accounting for approximately one-third of the high performance.

In solution 3, the study identifies a *dual EA–CD* strategy, revealing that provinces can achieve high CHI performance by implementing high EA and CD strategy when faced with not-high FS. The core conditions of solutions 2 and 3 are the same, which is referred to as a *second-order equivalent configuration*. The configuration has no other present conditions, indicating that the environment is essential to promoting CHI. The raw coverage of solution 3 is 0.42, the largest of the three, explaining a substantial share of the high CHI performance.

QCA regards correlation as the intersection of driving force and event result with asymmetrical characteristics [40]. Therefore, the configurations of not-high performance of CHI do not represent the negation of high-performance configurations, as is shown in Table 6.

Solution 4 represents a *TMC–EA–CD* strategy. Environmental conditions, EA and CD, are absent, indicating that the demand for CHI is weak, and the current social environment is unsuitable for CHI promotion. Although CD can be boosted by policy, it is difficult to enhance EA. With the presence of TI, the absence of TMC will affect the efficiency of technology promotion. The raw coverage of solution 4 is very low, at just 0.11, which explains the small-scale of not-high performance.

Solution 5 shows a *TI–EA* strategy. High GA implies that the government actively promotes CHI development by fully implementing relevant policies to allocate resources, such as tax incentives and financial subsidies; however, only exercising those authority behaviors cannot accomplish high performance without the presence of TI and EA. TI, the material carrier of practical technology applications is necessary, but has a relatively passive role in forming high-performance CHI. TI and EA are the most critical *hardware* and *software* required for technology promotion, which was revealed in the results of conditional necessity analysis (Table 4): EA and TI are the two conditions with the highest consistency (0.806 and 0.677), respectively. Solution 5 explains a significant proportion of the outcome, with raw coverage of 0.433.

Solution 6 is a *TI–TMC–EA* strategy, representing a second-order equivalent configuration of solution 5. The two technological conditions of TI and TMC are not present in this solution, indicating a serious inadequacy of technology supply. Such technical problems can primarily cause the outcome of not-high performance. High CD means that the technology has tremendous potential demand; however, due to the unsuitable social environment (not-high EA), this demand potential cannot be converted into normal demand. The solution's raw coverage is not large, at 0.164, explaining a small portion of the not-high CHI performance outcome.

Severe key implications are obtained through the comprehensive analysis of these six pathways. EA is present in three high-performance pathways and absent in all those with not-high performance. The study can conclude that EA is relatively more important than other conditions to achieving high CHI performance. This study reaches a conservative conclusion that it is not a necessary condition because EA's consistency, at 0.806, is below the threshold value of 0.9, and its effect size is just a little more than the threshold value of 0.1. There is no existing theory to justify considering EA as a necessary condition. FS does not have a positive role in all three high-performance pathways, and is even absent in the pathways; thus, FS has a negative effect on CHI promotion. A number of studies have examined the effect of FS on healthcare insurance, dividing the effect into three categories of positive, negative, and complex [66, 67, 68]. Previous studies were mainly based on conventional quantitative analysis, but research using QCA, such as this study, remains scarce.

To ensure the robustness of the sufficient condition analysis, this study increases the threshold values of consistency with a proportional reduction in inconsistency by 0.05; setting the two critical values to 0.85 and 0.8, respectively, repeating the analysis [69]. The resulting configurations in Table 7 should be a subset of the previous configurations. Solution 1 in Table 7 is a true subset of solution 1 in Table 6, and other solutions remain the same as those in Table 6, indicating that the analysis result is robust.

**Table 7:** Robustness check of configurations for achieving high and not-high CHI performance (fs/QCA).

Configuration	High performance			Not-high performance		
	1	2	3	4	5	6
TI	•			•	⊗	⊗
TMC	●			⊗		⊗
GA	•	•			•	
FS	⊗		⊗	●	●	●
EA	●	●	●	⊗	⊗	⊗
CD		●	●	⊗		•
Consistency	0.941	0.958	0.892	1	0.97	0.926
Raw coverage	0.196	0.293	0.42	0.11	0.433	0.164
Unique coverage	0.06	0.101	0.228	0.05	0.294	0.031
Overall solution consistency	0.907			0.975		
Overall solution coverage	0.581			0.519		

Comparing the various configurations of high-performance CHI, this study identifies the substitution relationships between conditions. First, a comparison of solutions 1 and 2 shows that TI plus TMC can be replaced with CD when GA and EA are simultaneously present. In addition, in a comparison of solutions 1 and 3, the combination of TI, TMC, and GA can be replaced with that of the negations of FS and CD with the presence of EA. Finally, comparing solution 2 with solution 3 reveals that GA can replace the negation of FS and vice versa when both EA and CD are simultaneously present.

The three substitutions support two perspectives. First, technological conditions (TI and TMC) and EA are relatively more important than other conditions. Technological conditions have strong flexibility in substitution relationships that can substitute organizational or environmental conditions under certain circumstances, in which the importance lies. Previous studies have shown that environmental conditions such as the education level of social residents, the degree of natural environment pollution, and organizational conditions such as economic policies and social security systems can affect the promotion of CHI [70, 71, 72, 73, 74]. The importance of EA focuses on establishing the three substitute relations based on the presence of the condition. Second, the negative effect of FS on the promotion of CHI is confirmed again. In addition, the effect has been proved by other studies [66, 67, 68]. That results from the substitution relationship that GA can replace each other with the negation of FS with the presence of EA and CD, and is consistent with the above analysis of sufficient condition.

## 4 Discussion and conclusion

There are three deficiencies in the current research about CHI. Firstly, the existing research focuses on the independent effect of one factor and lacks the consideration of the multi-factor synergistic effect of CHI promotion, which restricts the further understanding from the configuration perspective. Second, there is still a lack of research on promoting CHI using the technology–organization–environment theory from the perspective of technology adoption. As a means of risk management, CHI can be regarded as a technology to deal with risks, and purchasing CHI can be regarded as adopting and promoting technology. TOE theory believes that technology adoption is mainly and simultaneously affected by three factors: technology, organization, and environment. Therefore, the TOE framework is suitable for configuration analysis of the promotion of CHI. Finally, most existing literature using fs/QCA method does not remove the unreasonable hypothesis in the counterfactual analysis, so the results may deviate.

Given the above problems, this study innovatively constructs an analysis framework based on TOE theory. It uses fs/QCA to analyze how technology, organization, and environment constitute the promotion path of CHI of China from the perspective of configuration. In addition, the unreasonable hypothesis is removed in the counterfactual analysis. Three main conclusions are drawn. First, no conditions under the technology–organization–environment framework are necessary for high or not-high performance of CHI. However, there are three sufficient configurations to achieve high performance: the *technology–organization–environment* strategy, *government attention-environment adaptability-citizen demand* strategy, and dual *environment adaptability-citizen demand* strategy. In addition, there are three sufficient configurations to achieve the not-high performance, including *technological management capability-environment adaptability-citizen demand* strategy*, technological infrastructure-environment adaptability* strategy*,* and *technological infrastructure-technological management capability-environment adaptability* strategy. Second, TI, TMC, and EA are more important than the other conditions. The importance of the two technological conditions lies in the strong flexibility in substitution relationship that can substitute organizational or environmental conditions under certain circumstances. High EA is a prerequisite for high performance and these successful substitutions of conditions. Third, it is proved that the financial expenditure of government departments has a negative effect on the promotion of CHI.

The contribution of this study is concluded in the following three points. First, a new framework of fs/QCA is constructed to explore the paths driving high performance in the promotion of CHI from the perspective of configuration and the substitution relationship between the conditions. In addition, the driving paths of the non-high performance are investigated from the perspective of *causal asymmetry* [40]. The existing research regarding CHI only considers the independent effect of one factor, while examining the relationship between influential factors and CHI by regressions or other quantitative methods, whereas this study originally investigates such relationships through configuration analysis employing fs/QCA.

Second, the integrated analysis framework based on TOE theory with respect to technology adoption is constructed, which helps identify the driving path of the development of CHI from the perspective of technology adoption. This framework accounts for China's local characteristics, which helps better understand the influential factors of CHI. Thus, the intrinsic effect in the empirical research can be enhanced. Meanwhile, the scope of applying TOE theory is also extended. The TOE theory is usually used to determine the influential factors for the organizations' adoption of innovative technology [36]. It was also used to analyze the adoption of general information technology [38]. By expanding the technical connotation, other research objects can be analyzed as well by the framework proposed in this study, such as business model [39, 75], innovation decision [37], and usage of audit [76]. Introducing TOE into health insurance has an important theoretical contribution.

Third, the analysis from the configurations promotes the development of TOE theory. This study explores the concurrent synergistic effect and linkage matching mode of the multiple conditions (technology, organization, and environment) in the TOE framework regarding CHI. It further expands the application of the TOE framework to explain causal complexity. The TOE framework has been widely used to explain organizational technology adoption. However, most prior studies have only focused on the “net marginal effect” of a condition of technology, organization, or environment by applying statistical regression. Few studies have fully examined the linkage and matching of multiple conditions in the application of technology. Furthermore, the importance of the role of multiple conditions is rarely discussed in an integrative analytical framework. Nonetheless, this study helps to address the complexity of multiple conditions in the TOE framework. It can deepen researchers’ understanding of the complex mechanism behind the technology application.

The limitations of this study are as follows. Although the configuration analysis provides a better comprehensive and systematic understanding of the driving paths of CHI, it still has some limitations. First, calibration and counterfactual process in the QCA method is arbitrary. Therefore, the findings depending on researchers’ understanding of the research objects may be inaccurate. Second, this is the first time to apply QCA in the CHI study. A sensitivity analysis with other quantitative methods can further improve the robustness of methodological design. Third, with abduction logic, fs/QCA has the advantage of extending theory based on necessary and sufficiency causality [77, 78]. Such configurational contribution can be achieved in future studies using deduction logic to test causal complexity. Fourth, more observations of cases should be included in the investigation. The limitation of cases influences the conditions in fs/QCA analysis since possible configurations may be increased exponentially with the number of conditions contained. Future research can examine more complex settings involving additional cases, strategies, and operating conditions. Fifth, the temporal effect has not been considered in this study. All the cases employed are from the same year. So, it cannot reveal dynamism and turbulence. Therefore, the temporal effect in a QCA framework is a key point in future studies. Finally, as the first study to apply the technology adoption theory to CHI, there is no relevant theory to evaluate the adaptability of the TOE framework.

The study has some practical implications for governments. First, each region should choose a suitable driving path instead of making homogenization policies. In addition, the government should track the changes in the development level and conditions of CHI after implementing the policy and adjust the policy accordingly. Second, technological conditions and environmental adaptability as critical factors should be overcome. Technological conditions are relatively easy to achieve, but environment adaptability is more difficult; thus, the governments in areas of not-high EA should increase investment in social security to address residents' health risks. Third, although FS can crowd out CHI in some cases, it also has a pull effect. From the perspective of protecting the people's health, this should not be regarded as a competitive relationship, and the development of the two should be considered from a system's perspective. Therefore, governments should systematically evaluate social and CHI simultaneously and promote coordinated development.

## Declarations

### Author contribution statement

Xiuquan Huang: Conceived and designed the experiments; Wrote the paper.

Chih-Lin Tung: Performed the experiments.

Xi Wang, and Xiaocang Xu: Analyzed and interpreted the data.

Fat-Iam Lam and Tao Zhang: Contributed reagents, materials, analysis tools or data.

### Funding statement

Tao Zhang was supported by Macao Polytechnic Institute [RP/ESCHS-04/2020].

### Data availability statement

The data in this study are retrieved and compiled from the China Statistical Yearbook (http://www.stats.gov.cn/tjsj/ndsj/), the China Insurance Yearbook (https://data.cnki.net/trade/Yearbook/Single/N2011120065?zcode = Z016), and the China Stock Market & Accounting Research Database (https://cn.gtadata.com/).

### Declaration of interest's statement

The authors declare no conflict of interest.

### Additional information

No additional information is available for this paper.
